# A Robot Is Not Worth Another: Exploring Children’s Mental State Attribution to Different Humanoid Robots

**DOI:** 10.3389/fpsyg.2020.02011

**Published:** 2020-09-30

**Authors:** Federico Manzi, Giulia Peretti, Cinzia Di Dio, Angelo Cangelosi, Shoji Itakura, Takayuki Kanda, Hiroshi Ishiguro, Davide Massaro, Antonella Marchetti

**Affiliations:** ^1^Research Unit on Theory of Mind, Department of Psychology, Università Cattolica del Sacro Cuore, Milan, Italy; ^2^School of Computer Science, University of Manchester, Manchester, United Kingdom; ^3^Centre for Baby Science, Doshisha University, Kyoto, Japan; ^4^Human-Robot Interaction Laboratory, Graduate School of Informatics, Kyoto University, Kyoto, Japan; ^5^Advanced Telecommunications Research Institute International, IRC/HIL, Keihanna Science City, Kyoto, Japan; ^6^Department of Systems Innovation, Osaka University, Toyonaka, Japan

**Keywords:** child–robot interaction (cHRI), social robots, humanoid and anthropomorphic robots, differences among robots, children, anthropomorphism, mental states attribution

## Abstract

Recent technological developments in robotics has driven the design and production of different humanoid robots. Several studies have highlighted that the presence of human-like physical features could lead both adults and children to anthropomorphize the robots. In the present study we aimed to compare the attribution of mental states to two humanoid robots, NAO and Robovie, which differed in the degree of anthropomorphism. Children aged 5, 7, and 9 years were required to attribute mental states to the NAO robot, which presents more human-like characteristics compared to the Robovie robot, whose physical features look more mechanical. The results on mental state attribution as a function of children’s age and robot type showed that 5-year-olds have a greater tendency to anthropomorphize robots than older children, regardless of the type of robot. Moreover, the findings revealed that, although children aged 7 and 9 years attributed a certain degree of human-like mental features to both robots, they attributed greater mental states to NAO than Robovie compared to younger children. These results generally show that children tend to anthropomorphize humanoid robots that also present some mechanical characteristics, such as Robovie. Nevertheless, age-related differences showed that they should be endowed with physical characteristics closely resembling human ones to increase older children’s perception of human likeness. These findings have important implications for the design of robots, which also needs to consider the user’s target age, as well as for the generalizability issue of research findings that are commonly associated with the use of specific types of robots.

## Introduction

Currently, we are witnessing an increasing deployment of social robots (Bartneck and Forlizzi, 2004) in various contexts, from occupational to clinical to educational (Murashov et al., 2016; Belpaeme et al., 2018; Marchetti et al., in press). Humanoid social robots (HSRs), in particular, have proven to be effective social partners, possibly due to their physical human likeness (Dario et al., 2001). Humanoid social robots can vary in the degree of their anthropomorphic physical characteristics, often depending on the target user (children, adults, elderly, students, clinical populations, etc.) and the context (household, education, commercial, and rehabilitation). For example, the humanoid KASPAR robot that resembles a young child (with face, arms and hands, legs and feet), was specifically built for children with autism spectrum disorder (Dautenhahn et al., 2009; Wainer et al., 2014). In other instances, however, the same HSRs are used both for different purposes and different populations, like the NAO robot, which is largely used both with clinical and non-clinical populations (Shamsuddin et al., 2012; Mubin et al., 2013; Begum et al., 2016; Belpaeme et al., 2018), or the Robovie robot, that is employed both with adults and children (Shiomi et al., 2006; Kahn et al., 2012). A recent review of the literature by Marchetti et al. (2018) showed that different physical characteristics of HSRs may significantly affect the quality of interaction between humans and robots at different ages. The construction of robots that integrate and expand the specific biological abilities of our species led to two different directions in robotic development based on different, though related, theoretical perspectives: developmental cybernetics (DC; Itakura, 2008; Itakura et al., 2008; Moriguchi et al., 2011; Kannegiesser et al., 2015; Okanda et al., 2018; Di Dio et al., 2019; Wang et al., 2020; Manzi et al., 2020a) and developmental robotics (DR; De La Cruz et al., 2014; Cangelosi and Schlesinger, 2015, 2018; Lyon et al., 2016; Morse and Cangelosi, 2017; Vinanzi et al., 2019; Zhong et al., 2019; Di Dio et al., 2020a, b). The first perspective (DC) consists of creating a human-like system, by simulating human psychological processes and prosthetic functions in the robot (enhancing the function and lifestyle of persons) to observe people’s behavioral response toward the robot. The second perspective (DR) is related to the development of cognitive neural networks in the robot that would allow it to autonomously gain sensorimotor and mental capabilities with growing complexity, starting from intricate evolutionary principles. From these premises, the next two paragraphs briefly outline current findings concerning the effect that physical features of the HSRs have on human perception, thus outlining the phenomenon of anthropomorphism, and a recent methodology devised to measure it.

### Anthropomorphism

Anthropomorphism is a widely observed phenomenon in human–robot interaction (HRI; Fink et al., 2012; Airenti, 2015; Złotowski et al., 2015), and it is also greatly considered in the design of robots (Dario et al., 2001; Kiesler et al., 2008; Bartneck et al., 2009; Sharkey and Sharkey, 2011; Zanatto et al., 2016, 2020). In psychological terms, anthropomorphism is the tendency to attribute human characteristics, physical and/or psychological, to non-human agents (Duffy, 2003; Epley et al., 2007). Several studies have shown that humans may perceive non-anthropomorphic robots as anthropomorphic, such as Roomba (a vacuum cleaner with a semi-autonomous system; Fink et al., 2012). Although anthropomorphism seems to be a widespread phenomenon, the attribution of human traits to anthropomorphic robots is significantly greater compared to non-anthropomorphic robots. A study by Krach et al. (2008) compared four different agents (computer, functional robot, anthropomorphic robot, and human confederate) in a Prisoner’s Dilemma Game, and showed that the more the interactive partner displayed human-like characteristics, the more the participants appreciated the interaction and ascribed intelligence to the game partner. What characteristics of anthropomorphic robots (i.e., the HSRs) increase the perception of anthropomorphism? The HSRs can elicit the perception of anthropomorphism mainly at two levels: physical and behavioral (Marchetti et al., 2018). Working on the physical level is clearly easier than on intrinsic psychological features, and – although anthropomorphic physical features of robots are not the only answer to enhance the quality of interactions with humans – the implementation of these characteristics can positively affect HRIs (Duffy, 2003; for a review see Marchetti et al., 2018). It should be stated, however, that extreme human-likeness can result in the known uncanny valley effect, according to which HRIs are negatively influenced by robots that are too similar to the human (Mori, 1970; MacDorman and Ishiguro, 2006; Mori et al., 2012). Thus, the HSRs’ appearance represents an important social affordance for HRIs, as further demonstrated by the psychological research on racial and disability prejudice (Todd et al., 2011; Macdonald et al., 2017; Sarti et al., 2019; Manzi et al., 2020b). The anthropomorphic features of the HSRs can increase humans’ perception of humanness, such as mind attribution and personality, and influence other psychological mechanisms and processes (Kiesler and Goetz, 2002; MacDorman et al., 2005; Powers and Kiesler, 2006; Bartneck et al., 2008; Broadbent et al., 2013; Złotowski et al., 2015; Marchetti et al., 2020).

The study of the design of physical characteristics of the HSRs and their classification has been already investigated in HRI, but not systematically. A pioneering study by DiSalvo et al. (2002) explored the perception of humanness using 48 images of different heads of HSRs, and showed that three features are particularly important for the robot’s design: the nose, eyes, and mouth. Furthermore, a study by Duffy (2003) categorized different robots’ head in a diagram composed of three extremities: “human head” (as-close-as-possible to a human head), “iconic head” (a very minimum set of features) and “abstract head” (a more mechanistic design with minimal human-like aesthetics). Also, in this instance, human likeness was associated with greater mental abilities. Furthermore, a study by MacDorman (2006) analyzed the categorization of 14 types of robots (mainly androids and humanoids) in adults. It was shown that humanoid robots displaying some mechanical characteristics – such as the Robovie robot – were classified average on a “humanness” scale and rated lower on the uncanny valley scale. Recent studies compared one of the most widely used HSRs, the NAO robot, with different types of robots. It was shown that the NAO robot is perceived less human-like than an android – which is a highly anthropomorphic robot in both appearance and behavior (Broadbent, 2017)-, but more anthropomorphic than a mechanical robot, i.e., the Baxter robot (Yogeeswaran et al., 2016; Zanatto et al., 2019). However, there were no differences in perceived ability to perform physical and mental tasks between NAO and the android (Yogeeswaran et al., 2016), indicating that human-likeness (and not “human-exactness”) is sufficient to trigger the attribution of psychological features to a robot. In addition, a database has recently been created that collects more than 200 HSRs classified according to their level of human likeness (Phillips et al., 2018). In this study the NAO robot was classified with a score of about 45/100, in particular thanks to the characteristics of its face and body. Robovie and other similar robots were classified with a score ranging between 27 and 31/100, deriving mainly from body characteristics. These findings corroborate the hypothesis that NAO and Robovie are two HSRs with different levels of human-likeness due to their physical anthropomorphic features.

The interest in observing the effect of different physical characteristics of robots in terms of attribution of intentions, understanding, and emotions has also been investigated in children (Bumby and Dautenhahn, 1999; Woods et al., 2004; Woods, 2006). In particular, a study by Woods (2006), comparing 40 different robots, revealed that children experience greater discomfort with robots that look too similar to humans, favoring robots with mixed human-mechanical characteristics. These results were confirmed in a recent study by Tung (2016) showing that children preferred robots with not too many human-like features over robots with many human characteristics. Overall, these results suggested that an anthropomorphic design of HSRs may increase children’s preference toward them. Still, an excessive implementation of human features can negatively affect the attribution of positive qualities to the robot, again in line with the Uncanny Valley effect above.

### Attribution of Mental States

Different scales were developed to measure psychological anthropomorphism toward robots in adults. These scales typically assess attribution of intelligence, personality and emotions, only to mention a few. In particular, the attribution of internal states to the robot, i.e., to have a mind, is widely used and very promising in HRI (Broadbent et al., 2013; Stafford et al., 2014).

In psychology, the ability to ascribe mental states to others is defined as the Theory of Mind (ToM). Theory of mind is the ability to understand one’s own and others’ mental states (intentions, emotions, desires, beliefs), and to predict and interpret one’s own and others’ behaviors on the basis of such meta-representation (Premack and Woodruff, 1978; Wimmer and Perner, 1983; Perner and Wimmer, 1985). Theory of mind abilities develop around four years of age, becoming more sophisticated with development (Wellman et al., 2001). Theory of mind is active not only during humans’ relationships but also during interactions with robots (for a review, see Marchetti et al., 2018).

Recent studies have shown that adults tend to ascribe greater mental abilities to robots that have a human appearance (Hackel et al., 2014; Martini et al., 2016). This tendency to attribute human mental states to robots was also observed in children. Generally, children are inclined to anthropomorphize robots by attributing psychological and biological characteristics to them (Katayama et al., 2010; Okanda et al., 2019). Still, they do differentiate between humans and robots’ abilities. A pioneering study by Itakura (2008) investigating the attribution of mental verbs to a human and a robot showed that children did not attribute the epistemic verb “think” to the robot. More recent studies have further shown that already from three years of age, children fairly differentiate a human from a robot in terms of mental abilities (Di Dio et al., 2020a), although younger children appear to be more inclined to anthropomorphize robots compared to older children. This effect may be due to the phenomenon of animism, particularly active at three years of age (Di Dio et al., 2020a, b).

### Aim of the Study

The present study aimed to investigate the attribution of mental states (AMS) in children aged 5–9 years to two humanoid robots, NAO and Robovie, varying in their anthropomorphic physical features (DiSalvo et al., 2002; Duffy, 2003). Differences in the attribution of mental qualities to the two robots were then explored using the robots’ degree of physical anthropomorphism and the child’s chronological age. The two humanoid robots, NAO and Robovie, have been selected for two main reasons: (1) in relation to their physical appearance, both robots belong to the category of HSRs, but differ for their degree of anthropomorphism (for a detailed description of the robots, see section “Materials”); (2) both robots are largely used in experiments with children (Kanda et al., 2002; Kose and Yorganci, 2011; Kahn et al., 2012; Shamsuddin et al., 2012; Okumura et al., 2013a, b; Tielman et al., 2014; Cangelosi and Schlesinger, 2015, 2018; Hood et al., 2015; Di Dio et al., 2020a, b).

In light of previous findings associated with the use of these specific robots described above, we hypothesized the following: (1) independent of age, children would distinguish between humans and robots in terms of mental states by ascribing lower mental attributes to the robots; (2) children would tend to attribute greater mental qualities to NAO compared to Robovie because of its greater human-likeness; and (3) younger children would tend to attribute more human characteristics to robots (i.e., to anthropomorphize more) than older children.

## Materials and Methods

### Participants

Data were acquired on 189 Italian children from kindergarten and primary school age. The children were divided into three age groups for each robot as follows: (1) for the NAO robot, 5 years (*N* = 24, 13 females; *M* = 68.14; SD = 3.67); 7 years (*N* = 25, 13 females; *M* = 91.9; SD = 3.43); and 9 years (*N* = 23, 12 females; *M* = 116.38, SD = 3.91); (2) for the Robovie robot, 5 years (*N* = 33, 13 females; *M* = 70.9, SD = 2.95); 7 years (*N* = 49, 26 females; *M* = 93.4, SD = 3.62); and 9 years (*N* = 35, 15 females; *M* = 117.42, SD = 4.44). The initial inhomogeneity between sample sizes in the NAO and Robovie conditions were corrected by the random selection of children in the Robovie condition, caring to balance by gender. Accordingly, the sample for the Robovie condition used for statistical analysis was composed as follows: 5 years (*N* = 24, 8 females; *M* = 70.87, SD = 3.1); 7 years (*N* = 25, 14 females; *M* = 92.6, SD = 3.73); and 9 years (*N* = 23, 10 females; *M* = 117.43, SD = 4.62). The children’s parents received a written explanation of the procedure of the study, the measurement items, and gave their written consent. The children were not reported by teachers or parents for learning and/or socio-relational difficulties. The study was approved by the Local Ethic Committee (Università Cattolica del Sacro Cuore, Milan).

### Materials, Task, and Procedure

#### Materials

**FIGURE 1:**
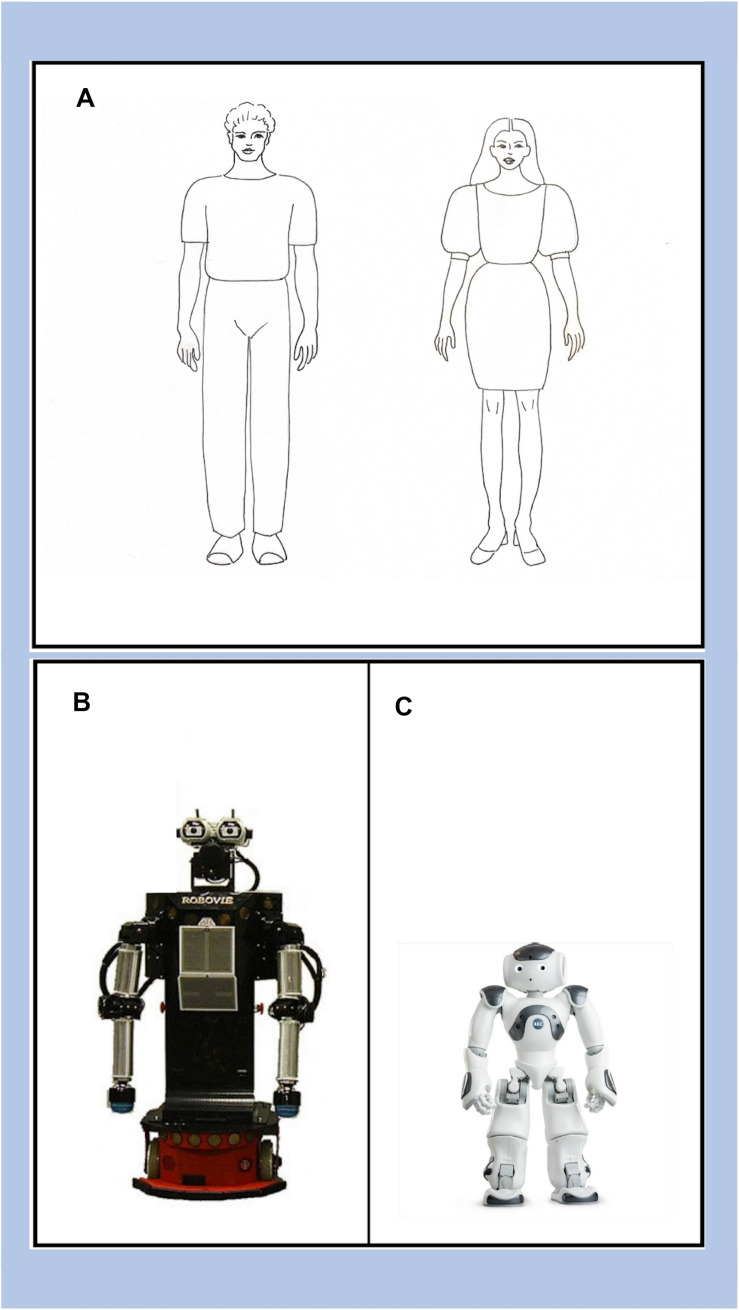
The AMS images: **(A)** the Human condition (male and female), **(B)** Robovie robot, and **(C)** the NAO robot.

The two HSRs selected for this study were the Robovie robot (Hiroshi Ishiguro Laboratories, ATR; Figure 1B) and the NAO robot (Aldebaran Robotics, Figure 1C). We chose these two robots because, although they both belong to the category of HSRs, they differ in their degree of anthropomorphic features (DiSalvo et al., 2002; Duffy, 2003; MacDorman, 2006; Zhang et al., 2008; Phillips et al., 2018). Robovie is a HSR with more abstract anthropomorphic features: no legs but two driving wheels to move, two arms without hands. In particular, the head can be considered “abstract” (Duffy, 2003) because of two important human-like features: two eyes and a microphone that looks like a mouth (DiSalvo et al., 2002). Robovie is an HSR that can be rated as average in the continuum of mechanical-humanlike (Ishiguro et al., 2001; Kanda et al., 2002; MacDorman, 2006). NAO is a HSR with more pronounced anthropomorphic features compared to Robovie: two legs, two arms, and two hands with three moving fingers (Figure 1C). Besides, the face can be classified as “iconic” and consists of three cameras suggesting two eyes and a mouth. However, considering the whole body and the more detailed shape of the face, NAO is a HSR that can be rated as more human-like than Robovie (DiSalvo et al., 2002; MacDorman, 2006; Phillips et al., 2018).

#### Attribution of Mental States

The AMS questionnaire^1^ is a measure of mental states that participants attribute to when they look at images depicting specific characters, in this case a human (female or male based on the participant’s gender; Figure 1A), and, according to the group condition, the Robovie or the NAO robot (Figures 1B,C). The AMS questionnaire was inspired by the methodology described in Martini et al. (2016) and is already used in several experiments with children (Manzi et al., 2017; Di Dio et al., 2019, 2020a, b). The construction of the content of the questionnaire is based on the theoretical model of Slaughter et al. (2009) on the categorization of children’s mental verbs resulting from communication exchanges between mother and child. This classification divides mental verbs into four categories: perceptive, volitional, cognitive, and dispositional. For the creation of the AMS questionnaire an additional category related to imaginative verbs has been added. We considered it necessary to distinguish between cognitive, epistemic, and imaginative states, since – especially for the robot – this specification enables the analysis of different psychological processes in terms of development. The AMS therefore consists of five dimensions: Perceptive, Emotive, Desires and Intentional, Imaginative, and Epistemic.

The human condition was used as a baseline measure to evaluate children’s ability to attribute mental states. In fact, as described in the results below, children scored quite high when ascribing mental attributes to the human character, thus supporting children’s competence in performing the mental states attribution task. Also, the human condition was used as a comparison measure against which the level of psychological anthropomorphism of NAO and Robovie was evaluated. The Cronbach’s alfa for each category is as follows: Perceptive (α = 0.8), Emotive (α = 0.8), Desires and Intentional (α = 0.8), Imaginative (α = 0.8), and Epistemic (α = 0.7).

Children answered 25 questions grouped into the five different state categories described above (see Appendix 1 for the specific items). The child had to answer “Yes” or “No” to each question, obtaining 1 when the response is “Yes” and 0 when the response is “No”. The sum of all responses (range = 0–25) gave the total score (α = 0.9); the five partial scores were the sum of the responses within each category (range = 0–5).

#### Procedure

The children were tested individually in a quiet room inside their school. Data acquisition was carried out by a single researcher during the normal school activities.

The experimenter showed each child the image on a paper depicting a human - gender matched - and one of the two robots, NAO or Robovie. The presentation order of the image -human and robot- was randomized. Afterward, the experimenter asked children the questions on the five categories of the AMS (Perceptive, Emotive, Intentions and Desires, Imaginative, and Epistemic). The presentation order of the five categories was also randomized. The total time required to complete the test was approximately 10 min.

## Results

### Data Analysis

To evaluate the effect of age, gender, states, agent, and type of robots on children’s mental state attribution to robots, a GLM analysis was carried out with five levels of *states* (Perceptive, Emotive, Intentions and Desires, Imaginative, and Epistemic) and two levels of *agent* (Human, Robot) as within-subjects factors, and *age* (5-, 7-, 9-year-olds), *gender* (Male, Female) and *robot* (Robovie, NAO) as the between-subjects factor. The Greenhouse-Geisser correction was used for violations of Mauchly’s Test of Sphericity (*p* < 0.05). *Post hoc* comparisons were Bonferroni corrected.

### Results

The results showed (1) a main effect of *agent*, *F*(1, 126) = 570.9, *p* < 0.001, *partial*-η^2^ = 0.819, δ = 1, indicating that children attributed greater mental states to the human (*M* = 4.6, SD = 0.27) compared to the robot (*M* = 2.7, SD = 0.21; *M*diff = 1.75, SE = 0.087); (2) a main effect of *states*, *F*(4,504) = 40.33, *p* < 0.001, *partial*-η^2^ = 0.243, δ = 1, mainly indicating that children attributed greater intention and desires and lower imaginative states (for a full description of the statistics, see Table 1); (3) a main effect of *robot*, *F*(1,126) = 39.4, *p* < 0.001, *partial*-η^2^ = 0.238, δ = 1, showing that children attributed greater mental states to NAO (*M* = 3.98, SD = 0.17) compared to Robovie (*M* = 3.4, SD = 0.14; *M*diff = 0.568, SE = 0.099).

**TABLE 1:** Statistics comparing the attribution of all AMS dimensions (Perceptive, Emotive, Intentions and Desires, Imaginative, Epistemic).

Dimension	Mental States	*M*diff	Err. Stan.	Sign.
Perceptive	Emotive	0.203	0.08	0.122
	Int&Des	**−0**.354*	0.071	**0.000**
	Imaginative	**0**.525*	0.075	**0.000**
	Epistemic	-0.089	0.069	1
Emotive	Perceptive	-0.203	0.08	0.122
	Int&Des	**−0**.557*	0.074	**0.000**
	Imaginative	**0**.322*	0.072	**0.000**
	Epistemic	**−0**.292*	0.079	**0.004**
Int&Des	Perceptive	**0**.354*	0.071	**0.000**
	Emotive	**0**.557*	0.074	**0.000**
	Imaginative	**0**.879*	0.071	**0.000**
	Epistemic	**0**.265*	0.067	**0.001**
Imaginative	Perceptive	**−0**.525*	0.065	**0.000**
	Emotive	**−0**.322*	0.070	**0.000**
	Int&Des	**−0**.879*	0.068	**0.000**
	Epistemic	**−0**.614*	0.074	**0.000**
Epistemic	Perceptive	0.089	0.065	**1**
	Emotive	**0**.292*	0.070	**0.004**
	Int&Des	**−0**.265*	0.068	**0.001**
	Imaginative	**0**.614*	0.074	**0.004**

Based on estimated marginal averages *The average difference is significant at the level of b Adaptation for multiple comparisons: Bonferroni. Significant values are in bold.

A two-way interaction was also found between (1) *states* and *agent, F*(1,126) = 16.51, *p* < 0.001, *partial*-η^2^ = 0.183, δ = 1 (for a detailed description of the differences see Table 2), and (2) *agent* and *age*, *F*(2,126) = 25.17, *p* < 0.001, *partial*-η^2^ = 0.285, δ = 1, showing that 5-year-old children attributed greater mental states to the robotic agents compared to older children (see Table 2).

**TABLE 2:** Statistics comparing the attribution of all AMS dimensions (Perceptive, Emotive, Intentions and Desires, Imaginative, Epistemic) and the AMS for the two agents (Human, Robot) across ages (5-, 7- and 9-years).

		Human			Robot		
	Age	*M*diff	Err. Stan.	Sign.	Mdiff	Err. Stan.	Sign.
	5 vs 7	**−0**.558*	0.103	**0**.**000**	**0**.443*	0.182	**0**.**05**
	5 vs 9	**−0**.558*	0.104	**0**.**000**	**0**.620*	0.183	**0**.**003**
	7 vs 9	-0.108	0.101	0.866	0.177	0.179	0.97

**State**	**Dimensions**	***M*diff**	**Err. Stan.**	**Sign.**	**Mdiff**	**Err. Stan.**	**Sign.**

Perceptive	Emotive	**0**.405*	0.202	**0**.**608**	7,63E-05	-0.351	0.351
	Int&Des	**0**.307*	0.121	**0**.**493**	**−0**.015*	-1.35	**−0**.**681**
	Imaginative	**0**.674*	0.458	**0**.**89**	**0**.376*	0.048	**0**.**704**
	Epistemic	**0**.218*	0.087	**0**.**35**	**−0**.396*	-0.758	**−0**.**035**
Emotive	Perceptive	**−0**.405*	-0.608	**−0**.**202**	−7,63E-05	-0.351	0.351
	Int&Des	-0.098	-0.323	0.126	**−1**.016*	-1.366	**−0**.**665**
	Imaginative	**0**.269*	0.046	**0**.**492**	**0**.376*	0.052	**0**.**7**
	Epistemic	-0.187	-0.401	0.028	**−0**.396*	-0.767	**−0**.**025**
Int&Des	Perceptive	**−0**.307*	-0.493	**−0**.**121**	**1**.015*	0.681	**1**.**35**
	Emotive	0.098	-0.126	0.323	**1**.016*	0.665	**1**.**366**
	Imaginative	**0**.367*	0.119	**0**.**616**	**1**.391*	1.078	**1**.**705**
	Epistemic	-0.088	-0.272	0.096	**0**.619*	0.296	**0**.**942**
Imaginative	Perceptive	**−0**.674*	-0.89	**−0**.**458**	−376*	-0.704	**−0**.**048**
	Emotive	**−0**.269*	-0.492	**−0**.**046**	**−0**.376*	-0.7	**−0**.**052**
	Int&Des	**−0**.367*	-0.616	**−0**.**119**	**−1**.391*	-1.705	**−1**.**078**
	Epistemic	**−0**.456*	-0.681	**−0**.**23**	**−0**.772*	-1.112	**−0**.**432**
Epistemic	Perceptive	**−0**.218*	-0.35	**−0**.**087**	**0**.396*	0.035	**0**.**758**
	Emotive	0.187	-0.028	0.401	**0**.396*	0.025	**0**.**767**
	Int&Des	0.088	-0.096	0.272	**−0**.619*	-0.942	**−0**.**296**
	Imaginative	**0**.456*	0.23	**0**.**681**	**0**.772*	0.432	**1**.**112**

Based on estimated marginal averages *The average difference is significant at the level of b Adaptation for multiple comparisons: Bonferroni. Significant values are in bold.

**FIGURE 2:**
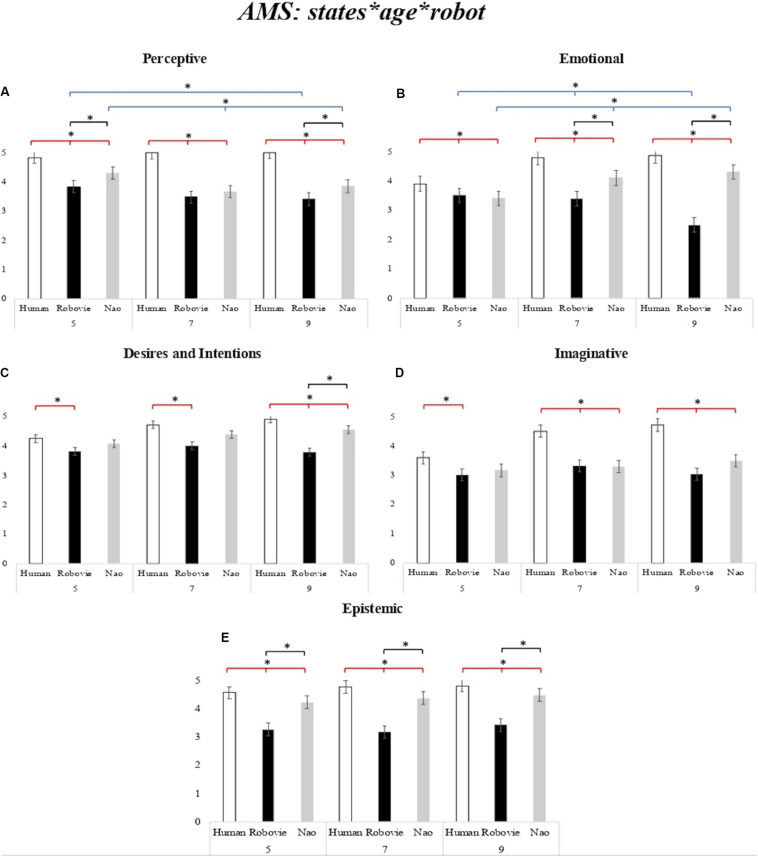
**(A–E)** Children’s scores on the attribution of mental states (AMS) scale. AMS mean scores for the Human (white bar), for Robovie robot (black bar), and NAO robot (gray bar) for each state (Perceptive, Emotions, Intentions and Desires, Imagination, and Epistemic) as a function of age group (5-, 7-, and 9-year-olds). The bars represent the standard error of the mean. *Indicates significant differences. The red lines indicate the differences between agents (Human, Robot); the blue lines indicate the differences between ages (5-, 7-, and 9-year-olds); the black lines indicate the differences between robots (Robovie, NAO).

Additionally, we found a three-way interaction between *states*, *age*, and *robot*, *F*(8,126) = 4.95, *p* < 0.001, *partial*-η^2^ = 0.073, δ = 1. The planned comparisons on the three-way interaction revealed that children attributed greater mental states to NAO compared to Robovie, with the youngest children differentiating on the Perceptive and Epistemic dimensions, and with this difference spreading to all dimensions (but imaginative) in the older children (see Figure 2).

## Discussion and Conclusion

### Discussion

In the present study we compared the AMS in children aged 5–9 years between two HSRs, NAO and Robovie, also with respect to a human. The aim was to explore children’s patterns of mental attribution to different types of HSRs, varying in their degree of physical anthropomorphism, from a developmental perspective.

Our results on the AMS to the human and robot generally confirmed the tendency of children to ascribe lower human mental qualities to the robots, thus supporting previous findings (Manzi et al., 2017; Di Dio et al., 2018, 2019, 2020a, b). In addition, children generally attributed greater mental states to the NAO robot than to the Robovie robot, although differences were found in the quality of mental states attribution as a function of age, with older children discriminating more between the types of robots that the younger ones. As a matter of fact, the important role played by the type of robot in influencing children’s AMS can be appreciated by evaluating differences in state attribution developmentally.

Firstly, 5-year-old children generally attributed greater human-like mental states to the robotic agents compared to older children. Additionally, while 5-year-old children discriminated between robots’ mental attribution only on the perceptive and epistemic dimensions – with the NAO robot being regarded as more anthropomorphic than Robovie –, children aged 7 and 9 years were particularly sensitive to the type of robots, and attributed greater mental states to NAO than Robovie on most of the tested mental state dimensions. From a developmental perspective, the tendency of younger children to anthropomorphize HSRs could be reasonably explained by the phenomenon of animism (Piaget, 1929). Already Piaget in 1929 suggested that children younger than 6 years tend to attribute a consciousness to objects, i.e., the phenomenon of animism, and that this fades around 9 years of age. Recently, this phenomenon has been defined as a cognitive error in children (Okanda et al., 2019), i.e., *animism error*, characterized by a lack of differentiation between living and non-living things. In this respect, several studies showed that, although children are generally able to discriminate between humans and robots, children aged 5–6 years tend to overuse animistic interpretations for inanimate things, and to attribute biological and psychological properties to robots (Katayama et al., 2010; Di Dio et al., 2019, 2020a, b), in line with the results of this study. Interestingly, we further found a difference in emotional attribution to NAO between 5-year-olds and 7- and 9-year-old children: younger children attributed lower emotions to NAO compared to the older ones. This result may seem counterintuitive in light of what we discussed above; however, by finely looking at the scores obtained from the 5-year-olds for each single emotional question, we found that younger children attributed significantly lower negative emotions to NAO compared to the other age groups, favoring positive emotions (χ^2^ < 0.01). This resulted in an overall decrease of scores in the emotional dimension for the young children. Therefore, not only does this result not contradict the idea of a greater tendency to anthropomorphize robots in younger children compared to older ones, but also highlights that 5-years-olds perceive NAO as a positive entity that cannot express negative emotions such as anger, sadness, and fear: the “good” play-partner.

From the age of 7, children’s belief of the robots’ mind is significantly affected by a sensitivity to the type of the robot, as shown by differences between NAO and Robovie on most mental dimensions, except for Imaginative. The lack of differences between robots on the Imaginative dimension (for all age-groups), which encompasses psychological processes like pretending, and making jokes, appears to be regarded by children as a human prerogative. Interestingly, this result supports findings from a previous study (Di Dio et al., 2018) that compared 6-year-old children’s mental state attribution to different entities (human, dog, robot, and God). Also, in that study, imagination was specific to the human entity.

Generally, the findings for older children indicate that the robot’s appearance does affect mental state attribution to the robot, and this is increasingly evident with age. However, the judgment of older children could also be significantly influenced by the robot’s behavioral characteristics, as demonstrated in a long-term study conducted with children aged 10–12 years (Ahmad et al., 2016). In this study, children played a snakes and ladders game with a NAO robot three times across 10 days, whose behavior in terms of personality for a social robot in education was adapted to maintain and create long-term engagement and acceptance. It was found that children positively reacted to the use of the robot in education, stressing a need to implement robots that are able to adapt based on previous experiences in real time. Of course, this is very much in line with the great vision of disciplines such DR (Cangelosi and Schlesinger, 2015) and DC (Itakura, 2008). In this respect, it is also important to consider further aspects related to the effectiveness in human relations of constructs such as understanding the perspective of others (e.g., Marchetti et al., 2018) and empathy, on which several research groups are actively working. For example, in an exploratory study Serholt et al. (2014) highlighted the perceived need both for teachers and learners to deal with robots showing such a competence.

In the same vein, other studies that used Robovie as an interactive partner in educational contexts, have also shown that when the robot is programed to facilitate interactional dynamics with children, it can be considered by the children as a group member and even part of the friendship circle. In these studies, the robot is typically programed to act as an effective social communicative partner using strategies, like calling children by their name, or adapting the interactive behaviors for each child by means of behavioral patterns drawn from developmental psychology (Kanda et al., 2007; see also, Kahn et al., 2012). The study by Kahn et al. (2012) further showed that after interacting with Robovie, most children believed that Robovie had mental states (e.g., was intelligent and had feelings) and was a social being (e.g., could be a friend, offer comfort, and be trusted with secrets).

The above studies highlight the prospective use of robots, particularly in the educational field. However, in reality, today’s robots are not yet able to sustain autonomous behavior in the long term, even though research is actively laying a good foundation for this. What we can certainly work on with direct effects on children’s perception of the mental abilities of robots are their physical attributes. By outlining differences in mental states attribution to different types of humanoid robots across ages based on robots’ physical appearance, our findings could help map the design of humanoid robots for children: in early ages, robots can display more abstract and mechanical features (possibly also due to the phenomenon of animism as described above); conversely, in older ages, the tendency to anthropomorphize robots is at least partially affected by the design of the robot. However, it has to be kept in mind that excessive human-likeness may be felt as uncomfortable, as suggested by findings showing that children experience less discomfort with robots displaying both human and mechanical features compared to robots whose physical features markedly evoke human ones (Bumby and Dautenhahn, 1999; Woods et al., 2004; Woods, 2006). Excessive resemblance to the human triggers the Uncanny Valley effect (the more the appearance of robots is similar to humans, the higher the sense of eeriness). These data suggest that a well-designed HSR for children should combine both human and mechanical dimensions, which, in our study, seems to be better represented by the NAO robot.

## Conclusion

This study enabled us to analyze the AMS to two types of HSRs, highlighting how different types of robot can evoke different attributions of mental states in children. More specifically, our findings suggest that children’s age is an important factor to consider when designing a robot, and provided us with at least two important insights associated with the phenomenon of anthropomorphism from a development perspective, and the design of HSRs for children. Anthropomorphism seems to be a widespread phenomenon in 5-year-olds, while it becomes more dependent on physical features of the robot in older children, with a preference ascribed to the NAO robot that is perceived as more human-like. This effect may then influence the design of robots, which can be more flexible in terms of physical features, as with Robovie, when targeted to young children.

Overall, our results suggest that the assessment of HSRs in terms of mental states attribution may represent a useful measure for studying the effect of different robots’ design for children. However, it has to be noted that the current results involved only two types of HSRs. Therefore, future studies will have to evaluate the mental attribution to a greater variety of robots by also comparing anthropomorphic and non-anthropomorphic robots, and across different cultures. In addition, in future studies it will be important to assess children’s socio-cognitive abilities such as language, executive functions, and ToM, to analyze the effect of these abilities on the AMS to robots developmentally. Finally, this study explored the mental attributions through images depicting robots. Future studies should include a condition where children interact with the robots *in vivo* to explore the intersectional effect between the robot’s physical appearance and its behavioral patterns. This would enable us to highlight the relative weight of each factor on children’s perception of the robots’ mental competences.

## Data Availability Statement

The datasets generated for this study are available on request to the corresponding author.

## Ethics Statement

The studies involving human participants were reviewed and approved by the Ethic Committee, Università Cattolica del Sacro Cuore, Milano, Italy. Written informed consent to participate in this study was provided by the participants’ legal guardian/next of kin.

## Author Contributions

All the authors conceived and designed the experiment. FM and GP conducted the experiments in schools. AM, FM, and GP secured ethical approval. FM and CDD carried out the statistical analyses. All authors contributed to the writing of the manuscript.

## Conflict of Interest

The authors declare that the research was conducted in the absence of any commercial or financial relationships that could be construed as a potential conflict of interest.

## Footnotes

1 http://www.teoriadellamente.it, “Strumenti” section

## Appendix 1

### Attribution of Mental States (AMS)

I will show you an image of a girl/boy/robot (to be selected depending on condition). I will ask you some questions about her/him/it (depending on condition). You can answer Yes or No to the questions.

**Table:**

**Dimensions (5) and questions (25)**
**Perceptive**
Do you think she/he/it can smell?
Do you think she/he/it can see?
Do you think she/he/it can taste?
Do you think she/he/it can hear?
Do you think she/he/it can feel hot or cold?
**Emotive**
Do you think she/he/it can get angry?
Do you think she/he/it can be scared?
Do you think she/he/it can be happy?
Do you think she/he/it can be surprised?
Do you think she/he/it can be sad?
**Intentions and desires**
Do you think she/he/it may have the intention to do something?
Do you think she/he/it might want to do something?
Do you think she/he/it might be willing to do something?
Do you think she/he/it can make a wish?
Do you think she/he/it might prefer one thing over another?
**Imaginative**
Do you think she/he/it can tell a lie?
Do you think she/he/it can pretend?
Do you think she/he/it can imagine?
Do you think she/he/it can make a joke?
Do you think she/he/it can dream?
**Epistemic**
Do you think she/he/it can understand?
Do you think she/he/it can make a decision?
Do you think she/he/it can learn?
Do you think she/he/it can teach?
Do you think she/he/it can think?

*http://www.teoriadellamente.it, “Strumenti” section.*
